# Photodynamic Activation of a Novel Chlorophyll-Enriched Green Propolis Compound Triggers Apoptosis in Renal Cell Carcinoma

**DOI:** 10.3390/ijms26146897

**Published:** 2025-07-18

**Authors:** Yao-Kuan Chen, Hui-Min Chiu, Shin-Yi Huang, Ta-Chun Liu, Daniel Tzu-Hsuan Chen

**Affiliations:** 1 Dr. Oxford Biotech Factory Co., Ltd., Taichung 407, Taiwan; 2 Oxperial Biohealth Ltd., Oxford OX2 6HT, UK; 3 Department of Oncology, University of Oxford, Oxford OX3 7DQ, UK; 4 Nuffield Department of Primary Care Health Science, University of Oxford, Oxford OX2 6GG, UK

**Keywords:** photodynamic therapy (PDT), Taiwanese green propolis, renal cancer, chlorophyll derivatives, nutraceuticals, apoptosis, GPDT

## Abstract

Renal cell carcinoma (RCC) presents significant therapeutic challenges due to its resistance to conventional treatments. Natural compounds with photodynamic properties, such as chlorophyll derivatives, offer potential for novel interventions. This study investigates the apoptotic effects of a chlorophyll-enriched green propolis compound activated by daylight-mediated photodynamic therapy (PDT) on RCC cells. A novel compound formulated from standardized ethanol extracts of Taiwanese green propolis, wheatgrass, and mulberry leaves was characterized using high-performance liquid chromatography (HPLC). Human RCC 786-O cells were treated with varying concentrations of the compound, with or without daylight PDT (570 nm). Cell viability was assessed via MTT assay, and median effective concentrations (EC_50_) were calculated. HPLC analysis identified Artepillin C as the major constituent. The compound induced dose-dependent cytotoxicity, which was significantly enhanced by daylight PDT. EC_50_ values dropped from 3.027 µL (compound alone) to 1.728 µL (with PDT), indicating synergistic efficacy. Cell viability significantly decreased in PDT-treated cells compared to non-treated controls (*p* < 0.05) indicating apoptosis. Daylight-activated PDT significantly amplifies the anticancer efficacy of the compound against RCC cells. Preliminary data suggest the potential of chlorophyll-enriched green propolis photodynamic activation (GPDT) as a natural adjunctive strategy for RCC, warranting further in vivo investigation.

## 1. Introduction

Cancer remains a leading cause of morbidity and mortality worldwide. Among various malignancies, renal cell carcinoma (RCC) accounts for approximately 90% of all kidney cancers and is characterized by resistance to conventional chemotherapy and radiotherapy [1]. Despite recent advancements in targeted therapies and immunotherapy, the prognosis for patients with advanced RCC remains poor [2], highlighting the urgent need for novel therapeutic approaches that are both effective and minimally toxic.

Natural compounds, especially those originating from medicinal plants and bee products, have been increasingly recognized for their potential as adjuncts in cancer prevention and therapy in recent decades [3]. These bioactive substances, often classified as nutraceuticals, offer a wide range of pharmacological benefits with relatively low toxicity profiles. Among them, Taiwanese green propolis, a resinous mixture collected by bees primarily from *Macaranga tanarius*, has gained significant attention due to its rich composition of polyphenolic compounds, notably flavonoids, phenolic acids, cinnamic acid derivatives, terpenes, fatty acids, and alkaloids [4,5,6]. More than 300 chemical constituents have been identified in various types of propolis, and their composition can vary depending on the botanical origin and geographic region [7].

Flavonoids within green propolis, such as quercetin, apigenin, and chrysin, have demonstrated strong pro-apoptotic and antiproliferative effects in a range of cancer cell lines, including prostate, breast, colon and oral cancers [8,9]. Mechanistically, these compounds promote mitochondrial-mediated apoptosis, inhibit angiogenesis, and modulate key oncogenic signalling pathways, such as NF-κB, PI3K/Akt, and MAPK pathways [10].

Similarly, chlorophyll-rich extracts from *Triticum aestivum* (wheatgrass) and *Morus alba* (mulberry leaves) were selected based on their well-documented pro-apoptotic, antioxidant, and photodynamic properties. These specific plants were chosen because they are not only rich in chlorophyll but also contain unique phytochemical profiles, such as sitosterol (in wheatgrass) and flavonoids like rutin and isoquercetin (in mulberry), which have demonstrated potent anticancer activities and shown superior compatibility and photodynamic activation in these two sources in previous studies [11,12,13]. Wheatgrass, abundant in chlorophyll, vitamins, and antioxidant enzymes, has been shown to induce oxidative stress selectively in cancer cells, thereby promoting apoptosis without harming normal cells [14]. Studies have highlighted its inhibitory effects on breast cancer, oral squamous cell carcinoma, and lung adenocarcinoma through mechanisms involving DNA damage induction and cell cycle arrest [15]. Mulberry leaf extracts, on the other hand, are rich in chlorophyll, flavonoids, and polyphenols, which exert cytotoxic effects against cervical, breast, and hepatocellular carcinoma cells by enhancing reactive oxygen species (ROS)-mediated apoptosis and disrupting mitochondrial membrane potential [16]. Collectively, these plant-derived and bee-derived nutraceuticals offer a multimodal approach to cancer management, combining antioxidant, anti-inflammatory, pro-apoptotic, and immunomodulatory effects, with promise as complementary agents in cancer prevention and therapy.

**Figure 1:**
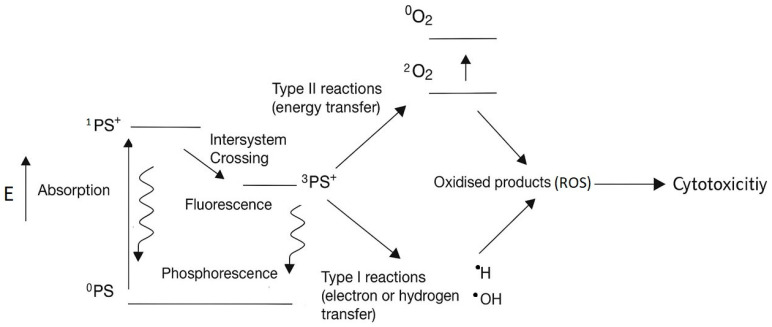
The mechanism of photodynamic therapy (PDT). By exposure to a light source (E), the photosensitizer (PS) absorbs energy and transitions into an excited singlet state (^1^PS^+^), followed by intersystem crossing to form an excited triplet state (^3^PS^+^). In the triplet state, the PS can engage in two distinct photochemical pathways: Type I reactions, where electron transfer to molecular oxygen leads to the formation of reactive oxygen species (ROS) including superoxide anions (O_2_•^−^), hydrogen peroxide (H_2_O_2_), and hydroxyl radicals (•OH), and Type II reactions, where energy transfer directly generates singlet oxygen (^1^O_2_). These ROS mediate oxidative stress, contributing to cytotoxicity and apoptosis. Abbreviations: PS, photosensitizer; ^1^PS^+^excited singlet state; ^3^PS^+^, excited triplet state; ROS, reactive oxygen species.

Recent innovations in nutraceutical science have aimed to enhance the therapeutic efficacy of natural products through formulation strategies such as chlorophyll enrichment and photodynamic therapy (PDT) [13]. PDT utilizes photosensitizers and light exposure to generate reactive oxygen species (ROS) via photochemical reactions, leading to oxidative stress and apoptosis in tumour cells (Figure 1) [17,18]. Advancements such as daylight PDT have gained interest for their non-invasive nature and broader clinical applicability, as they use natural or artificial light sources without requiring specialized equipment. In PDT, the photosensitizer is essential in initiating these reactions and is often used in combination with drugs to enhance therapeutic outcomes [19]. Notably, recent studies have shown that natural chlorophyll derivatives can effectively produce ROS, underscoring their potential as efficient and natural photosensitizers [20].

In this context, we developed a novel chlorophyll-enriched green propolis compound—comprising extracts from green propolis, wheatgrass, and mulberry leaves [21]—to assess its anticancer properties in combination with daylight PDT. Our previous parallel study demonstrated that such a compound can be activated by specific wavelengths of daylight to enhance anticancer efficacy in human glioblastoma cells [21]. Building on these findings, this study aims to evaluate the in vitro cytotoxic effects of the compound and to determine whether daylight-mediated photodynamic activation enhances its anticancer activity. By comparing treatment with and without daylight exposure, we seek to assess the photosensitizing potential of the compound and provide preliminary evidence supporting its future therapeutic development.

## 2. Results

### 2.1. Phytochemical Analysis of the Extract Compound by HPLC

**Figure 2:**
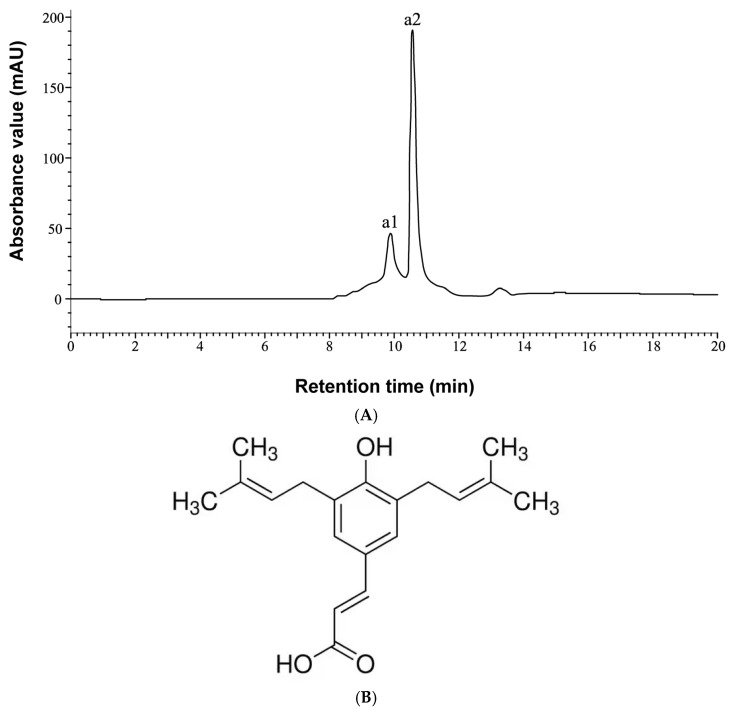
HPLC spectrum of the GP-WM extract and the chemical structure of Artepillin C. (**A**) HPLC separation of standard reference compounds from the GP–WM extract. Peaks a1 and a2 correspond to Artepillin C-related compounds. Detailed chromatographic conditions are provided in Table 1. (**B**) The chemical structure of Artepillin C.

To characterize the components of the extracts, high-performance liquid chromatography (HPLC) analysis was performed. The HPLC chromatogram of the GP-WM extract is presented in Figure 2. Two prominent peaks, designated as a1 and a2, were detected within the 20 min retention time. By comparison with standard reference compounds, both peaks were identified as Artepillin C, indicating that this compound is the predominant constituent in the GP-WM extract. Artepillin C, a bioactive molecule uniquely abundant in Taiwanese green propolis [22], has been reported to possess anticancer properties by inhibiting tumour cell proliferation and metastasis [23]. This study specifically investigated the apoptosis-inducing potential of the GP-WM extract.

### 2.2. Evaluation of the Effects of the GP-WM Extract on 786-O Cell Apoptosis with and Without Daylight PDT

**Figure 3:**
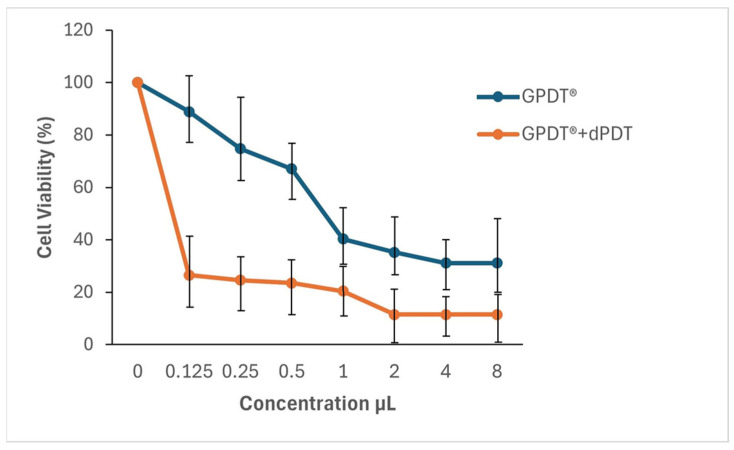
Effect of GP-WM extract on the viability of renal cell carcinoma 786-O cells with and without daylight photodynamic therapy (dPDT). The cells were treated with various concentrations (0.25, 0.5, 1, 2, 4, and 8 µL) of the GP-WM extract for 24 h and subsequent cell viability was measured via MTT assay. The data are presented in terms of proportional viability (%). Results represent the mean ± SE of two independent experiments, each performed with triplicate wells (*n* = 3) per concentration (0.25, 0.5, 1, 2, 4, and 8 µL), one set exposed to dPDT and the other unexposed. Statistical analysis was performed using one-way ANOVA followed by post hoc tests, with a significance threshold set at *p* < 0.05 to determine the statistical significance of the results. Abbreviations: dPDT: daylight photodynamic therapy.

The effects of the GP-WM extract on 786-O cell viability were assessed at various concentrations (0.25, 0.5, 1, 2, 4, and 8 µL) using the MTT assay, firstly without daylight photodynamic therapy (PDT). A clear dose-dependent reduction in cell viability was observed. Specifically, treatment with 0.125 µL of the GP-WM extract reduced viability from 100% to 88.9%; 0.25 µL reduced viability to 74.8%; 0.5 µL to 67.1%; 1 µL to 40.3%; 2 µL to 35.2%; 4 µL to 33.1%; and 8 µL to 31.1%. These results demonstrate that the GP-WM extract effectively reduced cell viability in 786-O cells in a concentration-dependent manner (Figure 3). To further quantify the inhibitory effects, the median effective concentration (EC_50_) was determined by sigmoidal curve fitting of the dose–response data. The EC_50_ of the GP-WM extract in 786-O cells, in the absence of daylight PDT, was calculated as 3.027 µL.

The combined effects of the GP-WM extract and daylight PDT were next evaluated using the MTT assay. Notably, the photodynamic treatment enhanced the inhibitory effect of the GP-WM extract on 786-O cells (Figure 3). A pronounced dose-dependent reduction in cell viability was also observed: treatment with 0.125 µL of the GP-WM extract under daylight PDT conditions reduced viability sharply from 100% to 26.5%; 0.25 µL reduced viability to 24.6%; 0.5 µL to 23.5%; 1 µL to 20.4%; 2 µL to 11.5%; and viability remained at 11.5% for both 4 µL and 8 µL doses. Following combination with daylight PDT, the EC_50_ of the GP-WM extract decreased markedly from 3.027 µL to 1.728 µL, reflecting improved efficacy.

Compared to the non-daylight PDT group, the GP-WM extract demonstrated significantly greater inhibition at concentrations of 0.25, 0.5, 1, and 2 µL (Figure 3), indicating that the combination treatment significantly improved cytotoxicity. These findings support the conclusion that photodynamic activation significantly potentiates the anticancer activity of the GP-WM extract against renal cancer cells.

## 3. Discussion

Natural and herbal compounds formulated as nutraceuticals have increasingly emerged as promising adjuncts in cancer management, including RCC. Natural products derived from plants and bee sources offer the advantage of multi-target biological activities, often accompanied by lower toxicity compared to conventional chemo- and radiotherapies. In vitro studies have shown that these compounds can inhibit tumour cell proliferation, induce apoptosis, prevent angiogenesis, and potentially enhance the effectiveness of existing therapies [3]. Their mechanisms of action, ranging from interference with oncogenic signalling pathways to chemical neutralization of carcinogens, highlight a broad spectrum of anticancer potential [10,24].

In this study, we evaluated the in vitro cytotoxic effects of a novel chlorophyll-enriched nutraceutical comprising ethanol GP-WM extracts from green propolis, wheatgrass, and mulberry leaves that leads to a reduction in cell viability, measured by MTT assay, suggesting cytotoxic effects that lead to apoptosis. Our results demonstrated that the extract of the compound effectively inhibited the viability of human renal carcinoma 786-O cells both with and without daylight PDT, indicating an apoptotic effect; however, the inhibitory effect was significantly amplified when combined with daylight PDT. While the reduction in cell viability is suggestive of apoptosis, further studies using apoptosis-specific assays (e.g., Annexin V/PI, caspase activity) are needed to clarify the mechanism of cell death. High-performance liquid chromatography (HPLC) analysis identified Artepillin C, a bioactive flavonoid abundant in green propolis, as a major constituent contributing to the observed anticancer activity [23,25]. Chlorophyll derivatives within the extract may have contributed to photodynamic effects due to their photosensitizing properties [20,26]. Other phytochemicals, such as β-sitosterol in wheatgrass [15], and flavonoids and alkaloids in mulberry leaves [16] may have further amplified the apoptotic effects observed, particularly when combined with daylight PDT.

The role of chlorophyll derivatives in improving PDT outcomes was further supported by our findings. Daylight PDT, which selectively targets malignant cells via photosensitizer activation, demonstrates synergistic effects when used alongside chlorophyll-rich compounds [26]. Previous studies have shown that combining green propolis with daylight PDT can increase cytotoxicity, induce apoptosis in cervical cancer cells [27], and enhance immune response [28]. Nonetheless, further investigations are necessary to identify and characterize the specific bioactive compounds within the extract that mediate these synergistic effects.

Importantly, our data demonstrate that chlorophyll-enriched green propolis photodynamic activation (GPDT) significantly reduces the viability of 786-O renal carcinoma cells in vitro under daylight exposure. This effect is consistent with our previous findings in human glioblastoma U87 cells [21]. The significant reduction in cell viability and lower EC_50_ values following combination therapy suggest an enhanced therapeutic potential. Despite these encouraging results, generalizability across different cell lines and translation to clinical applications will require comprehensive in vivo validation to assess pharmacokinetics, biodistribution, safety, and efficacy.

**Figure 4:**
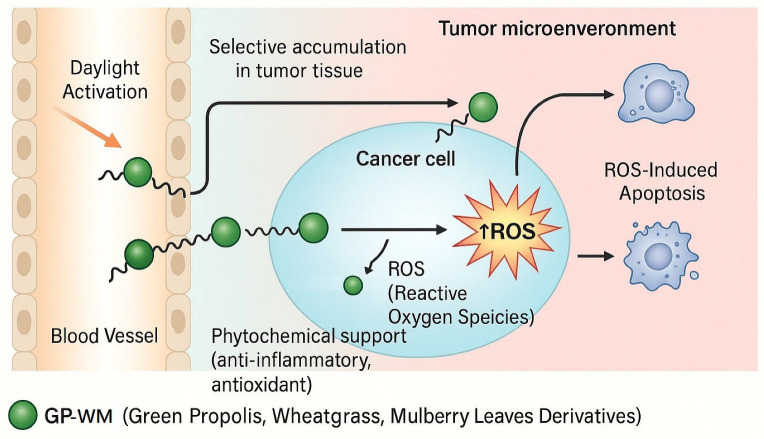
Diagram of the proprietary extract of GP-WM in targeting cancer cells. Conceptual representation illustrating the selective accumulation of GP-WM extracts of green propolis, wheatgrass, and mulberry leaf derivatives in the tumour microenvironment following systemic administration. Upon exposure to daylight (400–700 nm), the GP-WM molecules become photoactivated, generating reactive oxygen species (ROS) both extracellularly and intracellularly within cancer cells. The ROS production induces oxidative damage, triggering apoptosis selectively in tumour cells while minimizing injury to adjacent normal tissues. In addition to its photodynamic action, GP-WM provides phytochemical support through its intrinsic anti-inflammatory and antioxidant properties, contributing to an enhanced therapeutic profile.

Mechanistically, we hypothesize that upon daylight activation, the GP-WM extract acts as a photosensitizer, generating reactive oxygen species (ROS) that induce oxidative stress-mediated apoptosis in tumour cells (Figure 4). This concept aligns with our findings in glioblastoma U87 cells and previous studies demonstrating chlorophyll-based PDT efficacy [13,29]. However, ROS generation and downstream apoptotic pathways were not directly measured in the current study and warrant further investigation. Further mechanistic studies are required to confirm ROS production and specific cell death pathways, such as apoptosis, under these conditions.

While the preliminary in vitro findings are promising, as with many natural formulations, future studies must address several translational challenges. These include improving the systemic bioavailability and metabolic stability of the compound, determining its tissue distribution and photostability under physiological conditions, and minimizing potential phototoxicity to non-target tissues. Additionally, it is important to evaluate the selectivity of GP-WM-mediated photodynamic therapy toward cancer cells versus normal tissues, which may be influenced by tumour-specific uptake, redox status, and local microenvironment, which will be essential for optimizing the therapeutic window and progressing toward clinical translation.

## 4. Materials and Methods

### 4.1. Plant and Bee Product Sourcing

Green propolis was obtained from beehives maintained by Zhicheng Bee Farm (Wufeng District) and Mingyu Bee Farm (Waipu District), both located in Taichung City, Taiwan. Wheatgrass was cultivated in the greenhouse of Fang-Gwann Biotechnology Co., Ltd., situated in Mingjian Township, Nantou County, Taiwan. Mulberry leaves were sourced from the Quanming Ecological Education Sericulture Farm in Shitan Township, Miaoli County, Taiwan.

### 4.2. Cell Lines and Culture Conditions

The human renal cell carcinoma line 786-O (BCRC60243) was sourced from the Bioresource Collection and Research Centre (BCRC), affiliated with the Food Industry Research and Development Institute (FIRDI) in Hsinchu City, Taiwan. 786-O cells were maintained in sterile 10 cm polystyrene tissue culture-treated Petri dishes (Corning^®^ Cell Culture Dishes, surface-treated for adherent cell growth, Cat. No. 430167; Corning Inc., Corning, NY, USA) containing Dulbecco’s Modified Eagle’s Medium (DMEM, Gibco, Cat. No. 12100-038; Gibco, Thermo Fisher Scientific Inc., Waltham, MA, USA) supplemented with 10% fetal bovine serum (FBS) and 1% penicillin–streptomycin. Cultures were incubated at 37 °C in a 5% CO_2_ atmosphere. The culture medium was refreshed every two to three days, and cells were passaged when they reached 80% to 90% confluence.

### 4.3. Preparation of the Extract Compound

The extract was formulated based on the proportional weight of active compounds, specifically ethanol extracts derived from green propolis, wheatgrass, and mulberry leaves. Extraction and preparation protocols were documented in detail as follows.

#### 4.3.1. Green Propolis (GP) Extraction

To prepare the green propolis extract, propolis was combined with 95% ethanol in a 1:3 weight ratio and subjected to ultrasonication at temperatures between 30 °C and 50 °C for 1 h. After filtration, this ethanol extraction step was repeated three times. The resulting filtrates were pooled and vacuum-concentrated to yield a crude ethanol extract. This extract was then mixed with propylene glycol (1:1 ratio), ultrasonicated for 10 min, and vacuum-concentrated at 55 °C to 65 °C. The resulting propylene glycol solution was blended with medium-chain triglycerides (MCTs; derived from coconut oil, 60/40 caprylic/capric acid composition, purchased from Croda International Plc, Snaith, East Yorkshire, UK) in a 2:1 weight ratio, ultrasonicated again for 10 min, and separated using a thistle funnel. The lower layer, containing the dewaxed propylene glycol solution of green propolis, was collected as the GP extract.

#### 4.3.2. Wheatgrass and Mulberry Leaf (WM) Extraction

Dried wheatgrass (100 g) and dried mulberry leaves (100 g) were each extracted by mixing with 200 mL of 95% ethanol. The mixtures were ultrasonicated at 30 °C to 50 °C for 30 min and then filtered to obtain individual ethanol extracts. Equal volumes of both extracts were mixed (1:1 ratio) and allowed to stand at 25 °C for 36 h. The combined extract was filtered again, mixed with propylene glycol (1:1 ratio), and concentrated under vacuum to remove ethanol. The final propylene glycol solution containing both extracts was designated as the wheatgrass–mulberry (WM) extract.

#### 4.3.3. Composite GP-WM Extract Preparation

The GP and WM extracts were combined in equal proportions (1:1 ratio) to create a unified extract referred to as the GP-WM extract, which constitutes the proprietary blend of the study. This formulation was tested in a range of concentrations expressed as weight/weight ratios and evaluated in varying volumes (0, 0.25, 0.5, 1, 2, 4, and 8 µL) for experimental analyses.

### 4.4. Analyses of the Extract and Cell Viability

#### 4.4.1. High-Performance Liquid Chromatography (HPLC) Analysis

The GP-WM extract prepared for this study was analyzed by high-performance liquid chromatography (HPLC) at the Food Industry Research and Development Institute (FIRDI) in Taiwan. Detailed operating parameters and analytical conditions are provided in Table 1.

#### 4.4.2. Cell Viability Assay

Cell viability was evaluated using the MTT assay [30]. 786-O cells were seeded in 96-well plates at a density of 1 × 10^4^ cells per well and incubated with 100 μL of Dulbecco’s Modified Eagle’s Medium (DMEM) at 37 °C in a humidified atmosphere containing 5% CO_2_ for 24 h. After incubation, cells were treated with various concentrations of the GP-WM extract (0.25, 0.5, 1, 2, 4, and 8 µL) for 24 h.

Following treatment, one set of cell cultures was exposed to daylight photodynamic therapy (dPDT) using a 570 nm wavelength fluorescent LED lamp (maximum spectral output at 570 ± 10 nm; Everlight Electronics Co., Ltd., New Taipei City, Taiwan), positioned 25 cm above the 96-well plates. The light intensity was 9.5 mW/cm^2^, measured using a calibrated photodiode sensor, and the exposure duration was 24 h at room temperature in a darkened chamber to prevent ambient light interference. Control plates were shielded from light exposure. After 24 h, the medium was removed from all wells, and 5 mg/mL MTT solution was added. Cells were incubated again at 37 °C and 5% CO_2_ for 24 h. Subsequently, the MTT solution was removed, and the wells were washed twice with phosphate-buffered saline (PBS, pH 7.4). Then, 50 µL of dimethyl sulfoxide (DMSO) was added to each well to dissolve the formazan crystals, and absorbance was measured at 570 nm (OD_570_) using an ELISA reader (Synergy HTX Multi-Mode Reader, BioTek Instruments Inc., Winooski, VT, USA). The experiment was independently repeated in two experiments, each performed with triplicate wells (*n* = 3) for each concentration condition, both with and without dPDT exposure. Results represent the mean ± standard error (SE) of these two independent experiments. The median effective concentration (EC_50_) was calculated from the linear region of the dose–response curve.

#### 4.4.3. Statistical Analysis

Statistical analyses were conducted using GraphPad Prism 9 software. Data are presented as mean ± standard error (SE) for proportional viability (%) and EC_50_ values. One-way analysis of variance (ANOVA) was used to determine statistical significance, followed by post hoc testing when the F-statistic indicated *p* < 0.05 and no significant variance heterogeneity was detected. ANOVA was also employed for between-group comparisons (PDT-treated vs. non-treated) across different concentrations (0.25, 0.5, 1, 2, 4, and 8 µL). A *p*-value of <0.05 was considered statistically significant.

## 5. Conclusions

To our knowledge, this is among the first studies to report preliminary in vitro evidence of chlorophyll-enriched green propolis photodynamic activation (GPDT) exhibiting synergistic cytotoxicity with daylight-mediated PDT in RCC cells. While preliminary data suggest potential for future development, comprehensive mechanistic and in vivo validations are needed before such approaches can be considered for clinical application.

## 6. Patents

Patents for the invention based on the study have been granted in Taiwan (No: I806696) and the UK (No: GB2305047.9). Y.-K.C., H.-M.C and D.T.-H.C. declare that they are the patent holders of the invention based on this study.

## Figures and Tables

**Table 1:** The operating parameters and conditions for performing HPLC.

HPLC Instrument	Chromaster HPLC System, Equipped with a Pump (CM 5110) and a Diode Array Detector (CM 5430) (Hitachi High-Tech Corporation, Tokyo, Japan)
Type of chromatography column	C18 Column (Cosmosil^®^ 5C18-MS-II, Nacalai Tesque Inc., Kyoto, Japan)
Size of chromatography column	length: 250 mm inner diameter: 4.6 mm
Detection wavelength	320 nm
Mobile phase	Methanol (A)/0.4% phosphoric acid (B)(in methanol) (80:20, *v*/*v*)
Gradient elution	The mobile phase was conducted for 25 min as follows: A:B was 80:20 (*v*/*v*) for 0.1–9 min, A:B was 70:30 (*v*/*v*) for 9.1–12 min, A:B was 95:5 (*v*/*v*) for 12.1–15 min, A:B was 80:20 (*v*/*v*) for 15.1–25 min.
Flow rate of test sample	0.3 mL/min

## Data Availability

The datasets generated and/or analyzed during the current study are not publicly available due to their proprietary nature or ongoing analysis, but are available from the corresponding author on reasonable request.
